# Lymphoid Tissue–Resident *Alcaligenes* Establish an Intracellular Symbiotic Environment by Creating a Unique Energy Shift in Dendritic Cells

**DOI:** 10.3389/fmicb.2020.561005

**Published:** 2020-09-24

**Authors:** Koji Hosomi, Naoko Shibata, Atsushi Shimoyama, Tomoya Uto, Takahiro Nagatake, Yoko Tojima, Tomomi Nishino, Haruko Takeyama, Koichi Fukase, Hiroshi Kiyono, Jun Kunisawa

**Affiliations:** ^1^Laboratory of Vaccine Materials, Center for Vaccine and Adjuvant Research, Ibaraki, Japan; ^2^Laboratory of Gut Environmental System, National Institutes of Biomedical Innovation, Health, and Nutrition (NIBIOHN), Ibaraki, Japan; ^3^International Research and Development Center for Mucosal Vaccines, The Institute of Medical Science, The University of Tokyo, Tokyo, Japan; ^4^Research Organization for Nano and Life Innovation, Waseda University, Tokyo, Japan; ^5^Graduate School of Science, Osaka University, Toyonaka, Japan; ^6^Department of Life Science and Medical Bioscience, Waseda University, Tokyo, Japan; ^7^IMSUT Distinguished Professor Unit, The Institute of Medical Science, The University of Tokyo, Tokyo, Japan; ^8^Graduate School of Medicine, Chiba University, Chuo City, Japan; ^9^Department of Medicine, School of Medicine and Chiba University – UC San Diego Center for Mucosal Immunology, Allergy, and Vaccine, University of California, San Diego, San Diego, CA, United States; ^10^Graduate School of Medicine, Osaka University, Suita, Japan; ^11^Graduate School of Pharmaceutical Sciences, Osaka University, Suita, Japan; ^12^Graduate School of Density, Osaka University, Suita, Japan; ^13^Graduate School of Medicine, Kobe University, Kobe, Japan

**Keywords:** inducible nitric oxide synmase, lipopolysaccharid, mitochondrial respiration, lymphoid-tissue-resident commensal bacteria, apoptosis, dendritic cells

## Abstract

Lymphoid-tissue–resident commensal bacteria (LRCs), including *Alcaligenes faecalis*, are present in intestinal lymphoid tissue including the Peyer’s patches (PPs) of mammals and modulate the host immune system. Although LRCs can colonize within dendritic cells (DCs), the mechanisms through which LRCs persist in DCs and the symbiotic relationships between LRCs and DCs remain to be investigated. Here, we show an intracellular symbiotic system in which the LRC *Alcaligenes* creates a unique energy shift in DCs. Whereas DCs showed low mitochondrial respiration when they were co-cultured with *Escherichia coli*, DCs carrying *A. faecalis* maintained increased mitochondrial respiration. Furthermore, *E. coli* induced apoptosis of DCs but *A. faecalis* did not. Regarding an underlying mechanism, *A. faecalis*—unlike *E. coli*—did not induce intracellular nitric oxide (NO) production in DCs due to the low activity of its lipopolysaccharide (LPS). Therefore, *A. faecalis*, an example of LRCs, may persist within intestinal lymphoid tissue because they elicit little NO production in DCs. In addition, the symbiotic DCs exhibit characteristic physiologic changes, including a low rate of apoptosis and increased mitochondrial respiration.

## Introduction

The gut microbiota has garnered attention recently because these organisms exert several biologic effects, including the development and regulation of the host immune system, and are associated with health maintenance and the risk for several diseases (Hooper and Macpherson, 2010; Cani, 2018). Most intestinal commensal bacteria are located in the lumen of the intestine and are obligate anaerobic bacteria belonging to the phyla Firmicutes and Bacteroidetes (Hooper and Macpherson, 2010). Recent studies using 16S rDNA sequencing and fluorescent *in situ* hybridization analysis have revealed unique bacterial communities in intestinal lymphoid tissue, including Peyer’s patches (PPs), lymphoid follicles, and the mesenteric lymph nodes (mLNs) of mice, non-human primates, and humans (Obata et al., 2010). These lymphoid-tissue–resident commensal bacteria (LRCs) belong to the α- and β-proteobacteria groups, which are facultatively anaerobic and can survive or grow in an oxygenated environment (Fung et al., 2016). Indeed, LRCs DNA have been detected in CD11c^+^ DCs in the PPs and mLNs of mice and efficiently colonize and persist in murine DCs (Obata et al., 2010). Within the PPs of mice, *Alcaligenes* spp., including *A. faecalis*, are a dominant genus among LRCs (Obata et al., 2010). Furthermore, after being taken up by M cells, *A. faecalis* is captured by CD11c^+^ DCs in PPs (Sato et al., 2013; Shibata et al., 2018). Indeed, depletion of CD11c^+^ DCs leads to the absence of *A. faecalis* in the PPs of mice (Shibata et al., 2018).

Like lumen-resident commensal bacteria, LRCs modulate the host immune system and are involved in the inflammation associated with chronic diseases. The depletion of innate lymphoid cells (ILCs) resulted in peripheral dissemination of LRCs and systemic inflammation in mice (Sonnenberg et al., 2012). In addition, *Alcaligenes*-specific systemic immune responses are associated with Crohn’s disease and progressive hepatitis C virus infection (Sonnenberg et al., 2012). LRCs induced multiple members of the IL-10 cytokine family, including DC-derived IL-10, and ILC3-derived IL-22, and provided protective effects in a murine colitis model (Fung et al., 2016). Moreover, we previously demonstrated that *A. faecalis* activates DCs to produce IL-6 through the weak agonistic activity of its lipopolysaccharide (LPS) against toll-like receptor (TLR) 4 together with low inflammatory activity, indicating that *Alcaligenes* spp. maintain their homeostatic environment in PPs without inducing an excessive inflammatory response (Shibata et al., 2018). These evidences highlight that LRCs, including *A. faecalis*, interact directly with DCs in intestinal lymphoid tissue and regulate their immune functions to maintain immunologic homeostasis in the intestine. Despite these findings, the mechanisms through which LRCs persist in DCs and the symbiotic relationships between LRCs and DCs remain to be investigated.

Nitric oxide (NO) plays a crucial role in the antimicrobial activity of the host immune defense system, and through the action of inducible NO synthetase (iNOS), DCs produce NO for killing invading bacteria (Bogdan, 2015). In addition, NO acts as a signaling molecule and has several biologic functions, including the regulation of energy metabolism and apoptosis (Erusalimsky and Moncada, 2007). The energy metabolism of immune cells varies depending on their differentiation and activation and is involved in the regulation of their functions (Pearce and Pearce, 2013). For example, M1 macrophages, which play an important role in the clearance of microbial infections and are characterized by their production of inflammatory cytokines and NO, exhibit high levels of glycolysis and low oxidative phosphorylation in mitochondria (Mills and O’Neill, 2016).

In the current study, we found that, because of its low LPS activity, *A. faecalis* had a weak activity to induce NO elevation with low expression of iNOS in the DCs. In addition, DCs carrying *A. faecalis* show a low rate of apoptosis and a unique shift in energy metabolism, that is, increased mitochondrial respiration. These findings further our understanding of the symbiotic relationships between LRCs, including *Alcaligenes* spp., and intestinal lymphoid tissue.

## Materials and Methods

### Mice

Female C57BL/6 mice (age, 4 weeks) were purchased from Japan SLC (Shizuoka, Japan) for experiments. All experiments were approved by the Animal Care and Use Committee of the National Institutes of Biomedical Innovation, Health, and Nutrition (approval no. DS27-48R10) and were conducted in accordance with their guidelines.

### Bacteria Culture and LPS Preparation

*Alcaligenes faecalis* (13111T) and *Escherichia coli* (IID561) were obtained from the National Institute of Technology and Evaluation Biological Resource Center (Osaka, Japan) and the Pathogenic Microbes Repository Unit (Tokyo, Japan), respectively. *A. faecalis* and *E. coli* were cultured aerobically in LB broth (Nacalai Tesque, Kyoto, Japan) at 37°C. Bacterial LPS was extracted from lyophilized *A. faecalis* and *E. coli* by using an LPS Extraction Kit (iNtRON Biotechnology, Sangdaewon-Dong, Korea) according to the manufacturer’s instructions. The extracted LPS was solved in PBS to a concentration of 1 mg/ml and stored at –30°C until use.

### Bone Marrow-Derived Dendritic Cells

Bone marrow-derived dendritic cells (BMDCs) were prepared as previously described (Sato et al., 2006). Briefly, BM was collected from the femurs of mice and cultured in RPMI 1640 containing 10% fetal bovine serum (FBS), 55 μM 2-mercapethanol, 1 mM pyruvate, 1% penicillin–streptomycin, and 20 ng/ml granulocyte macrophage colony stimulating factor (GM-CSF) at 37°C in 5% CO_2_ for 6 days; half of the medium was replaced with fresh every other day. After 6 days, BMDCs were purified by using anti-mouse CD11c magnetic beads and a Magnetic Cell Separation System (MACS) (Miltenyi Biotech, Bergisch Gladbach, Germany) according to the manufacturer’s protocols.

### Co-culture of BMDCs With Live Bacteria

BMDCs were seeded in 96-well plates at 1 × 10^5^ cells/well or 24-well plates at 5 × 10^5^ cells/well in RPMI 1640 containing 10% FBS, 2-mercapethanol, pyruvate, and penicillin–streptomycin and cultured for 8–16 h to allow cells to attach to plates. After they were washed with RPMI 1640 medium containing 10% FBS, 2-mercapethanol, and pyruvate but without antibiotics, BMDCs were co-cultured with live *A. faecalis* or *E. coli* at 10 multiplicity of infection (MOI) for 1 h and then washed with fresh media containing 100 μg/ml gentamycin to kill and remove extracellular bacteria. After incubation in RPMI 1640 containing 10% FBS, 2-mercapethanol, pyruvate, and 100 μg/ml gentamycin, BMDCs were used for several experiments. S-ethyl isothiourea (SEIT) (Cayman Chemical, MI, United States) was dissolved in ethanol (Nacalai Tesque) at 150 mM for stock solution and used at final concentration of 500 μM diluted with culture medium as iNOS inhibitor. All cell incubations were performed at 37°C in 5% CO_2_.

### Reverse Transcription and Quantitative PCR Analysis (RT-qPCR)

Reverse transcription and quantitative PCR analysis was performed as previously described (Nagatake et al., 2019). Total RNA was isolated from BMDCs by using Sepazol (Nacalai Tesque) and chloroform (Nacalai Tesque), precipitated with 2-propanol (Nacalai Tesque), and washed with 75% (vol/vol) ethanol (Nacalai Tesque). RNA samples were incubated with DNase I (Invitrogen, Carlsbad, CA, United States) to remove contaminating genomic DNA and then were reverse-transcribed into cDNA (Superscript III reverse transcriptase, VIRO cDNA Synthesis Kit, Invitrogen). Quantitative PCR analysis was performed by using a LightCycler 480 II system (Roche, Basel, Switzerland) with FastStart Essential DNA Probes Master (Roche). Primer sequences were 5′-ctttgccacggacgagac-3′ and 5′-tcattgtactctgagggctgac-3′ for *Nos2*, 5′-caaggaggccatcaatgtg-3′ and 5′-gggaattcattgtacagttccac-3′ for *Rab7*, 5′-cctacgagactgcgaatggt-3′ and 5′-ccacaagaactgccatttttc-3′ for *Lamp-1*, and 5′-aaggccaaccgtgaaaagat-3′ and 5′-gtggtacgaccagaggcatac-3′ for *Actinb*.

### Flow Cytometric Analysis (FACS)

Flow cytometry was performed as previously described (Nagatake et al., 2018). BMDCs were collected by using trypsin–EDTA solution (Nacalai Tesque) and suspended in PBS containing 2% (vol/vol) newborn calf serum (Equitech-Bio, Kerrville, TX, United States). The cells were stained with an anti-CD16/32 monoclonal antibody (mAb) (TruStain fcX, Biolegend, clone 93) and 7-AAD (Biolegend) to avoid non-specific staining and detect dead cells, respectively, for 15 min at room temperature. The cells were further stained with the fluorescently labeled Ab BV421-anti-MHCII (Biolegend, clone M5/114.15.2). To detect apoptotic cells, the cells were stained with APC Annexin V (Biolegend) according to the manufacturer’s protocols. For intracellular FACS, the cells were stained with Zombie (Biolegend) to detect dead cells. The cells were further stained with PE-anti-iNOS (eBioscience, clone CXNFT) after their treatment with BD Cytofix/Cytoperm Fixation and Permeabilization Solution (BD Biosciences, Franklin Lakes, NJ, United Sates) according to the manufacturer’s protocols. To examine mitochondrial membrane potential (Δψm), the cells were stained with tetramethylrhodamine methyl ester (Thermo Fisher Scientific, Tokyo, Japan), and fluorescence intensity was measured immediately after incubation for 15 min. Samples were analyzed by using MACSQuant (Miltenyi Biotech, Bergish Gladbach, Germany), and data analysis was performed by using FlowJo 9.9 (Tree Star, Ashland, OR, United States).

### Measurement of Intracellular NO

Intracellular NO was measured by using an OxiSelect Intacellular Nitric Oxide Assay Kit (Cell Biolabs) according to the manufacturer’s protocols. After BMDCs were co-cultured with live bacteria or incubated with bacterial LPS in the presence of 10 ng/ml IFN-γ, the cells were stained with NO probe, and the fluorescence was measured by using a multimodal plate reader (ARVO X2, PerkinElmer, Waltham, MA, United States), at wavelengths of 485 nm (excitation) and 530 nm (emission).

### NO Degrading Analysis

*A. faecalis* was suspended in RPMI 1640 medium and seeded in 96-well plates at 1 × 10^8^, 3 × 10^8^, 1 × 10^9^ CFU/well. (±)-(E)-4-Ethyl-2-[(E)-hydroxyimino]-5-nitro-3-hexenamide (NOR3) (Dojindo Lab., Kumamoto, Japan) was added into the well at the concentration of 100 μM and the mixture was incubated at 37°C for 1 h. After the incubation, the concentration of NO was measured by Griess reagent kit (Invitrogen) according to the manufacturer’s protocols.

### Flux Analysis

Basal oxygen consumption rate (OCR) and extracellular acidification rate (ECAR) were measured by using a flux analyzer (Seahorse Bioscience XF24 Extracellular Flux Analyzer, Agilent, Santa Clara, CA, United States) and XF Mito Stress Kit (Agilent). BMDCs (1 × 10^5^ cells/well) were plated on Seaphorse 24-well plates, and basal levels of oxygen consumption and proton production were quantified over 20–25 min. Then, rotenone and antimycin A were injected to inhibit mitochondrial complexes I and III, leading to cessation of both electron flow and oxygen. Experiments were performed by measuring 3 simultaneous replicates of each well. Basal respiration rates were calculated by using the manufacturer-provided software.

### Statistics

Statistical analyses were performed by using Prism 7 (GraphPad Software, CA, United States). Data are presented as mean ± SD. Significant differences among two or more than three groups were calculated using the Student’s *t-*test or one-way ANOVA test, respectively (^∗^*P* < 0.05; ^∗∗^*P* < 0.01; n.s., not significant).

### Data Availability Statements

The raw data supporting the conclusions of this article will be made available by the authors, without undue reservation.

## Results

### Low Expression of *Nos2* in DCs Carrying *A. faecalis*

Our previous study demonstrated that LRCs including *A. faecalis* survive longer in DCs than do luminal-resident bacteria including *E. coli* (Fung et al., 2016). To gain insight into the mechanism through which this symbiosis is established, we examined the gene expression of some factors involved in the elimination of intracellular bacteria, including NO production (*Nos2*) and phagocytosis (*Rab7* and *Lamp-1*) and expression of major histocompatibility complex (MHC) class II (MHCII) to evaluate activation status of BMDCs. The expressions of *Rab7* and *Lamp-1* were similarly reduced in BMDCs carrying either *A. faecalis* or *E. coli* in comparison with non-stimulated BMDCs (Supplementary Figure 1A). Similar increase of MHCII expression was noted in BMDCs carrying either *A. faecalis* or *E. coli* in comparison with non-stimulated BMDCs (Supplementary Figure 1B). Thus, similar changes were observed in markers for phagocytosis and cell activation when they were co-cultured with *A. faecalis* or *E. coli*.

**FIGURE 1:**
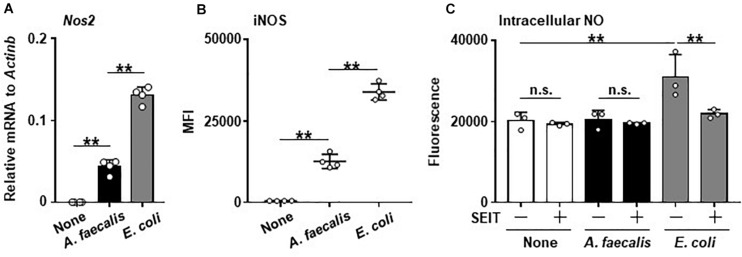
Low levels of iNOS expression and little intracellular NO production in DCs induced by *A. faecalis.* BMDCs were co-cultured without (None) or with live *A. faecalis* or *E. coli* at 10 MOI for 24 h, after which *Nos2* gene expression by RT-qPCR **(A)**, iNOS expression **(B)**, and intracellular NO **(C)** were measured in the presence or absence of the iNOS inhibitor SEIT. To measure iNOS expression by intracellular FACS, after the co-culture, BMDCs were collected, stained with PE-anti-iNOS mAb after treatment of BD Cytofix/Cytoperm Fixation and Permeabilization Solution, and analyzed by flow cytometry. Measurement of intracellular NO was performed using an OxiSelect Intacellular Nitric Oxide Assay Kit (Cell Biolabs). After the co-culture, BMDCs were stained with NO probe and the fluorescence was measured. The results shown are representative of two independent experiments. *P < 0.05; **P < 0.01; n.s., not significant, which is described in Materials and Methods.

Expression of *Nos2* was greatly increased in BMDCs carrying *E. coli* compared with non-stimulated BMDCs. Similarly, the treatment of BMDCs with *A. faecalis* induced the *Nos2* expression, but its level was significantly lower than that in BMDCs carrying *E. coli* (Figure 1A). Thus, these results indicate that *A. faecalis* has a lower activity to induce *Nos2* expression than *E. coli* in DCs.

### Little Induction of Intracellular NO in DCs by *A. faecalis*

Consistent with the gene expression analysis, the expression of *Nos2*-encoded iNOS was highly increased in BMDCs carrying *E. coli*, but the iNOS expression level was significantly lower in BMDCs carrying *A. faecalis* than in BMDCs harboring *E. coli* (Figure 1B and Supplementary Figure 2). Furthermore, when we examined intracellular NO production, BMDCs carrying *E. coli* had more intracellular NO than either non-stimulated BMDCs or BMDCs carrying *A. faecalis* (Figure 1C). Treatment with the iNOS inhibitor SEIT suppressed intracellular NO elevation in BMDCs carrying *E. coli*, indicating that the observed NO production was iNOS-dependent (Figure 1C). In contrast, NO level did not differ between non-stimulated BMDCs and BMDCs carrying *A. faecalis* (Figure 1C). Additionally, we found that *A. faecalis* possess NO degrading activity. Indeed, NO induced by NOR3, a NO inducing reagent, was reduced by incubation with *A. faecalis* in a dose dependent manner (Supplementary Figure 3). Thus, *A. faecalis* has dual functions including low activity to induce intracellular NO production and NO degradation.

### Low Activity of *A. faecalis*-Derived LPS for Producing Intracellular NO in DCs

**FIGURE 2:**
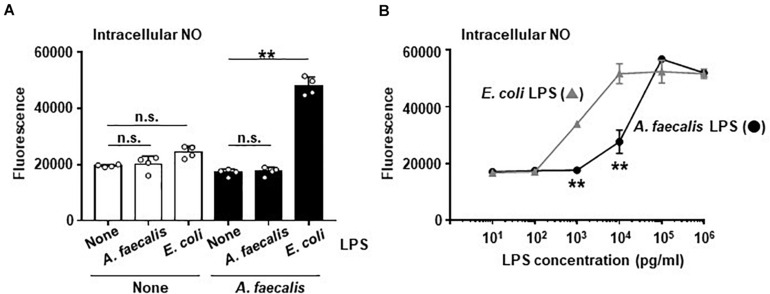
Low activity of *A. faecalis* LPS to produce intracellular NO in DCs. **(A)** BMDCs were co-cultured without (None) or with live *A. faecalis* at 10 MOI in the absence (None) or presence of *A. faecalis* LPS or *E. coli* LPS at 0.1 μg/ml for 24 h. **(B)** BMDCs were incubated with *A. faecalis* LPS or *E. coli* LPS in the presence of IFN-γ at 10 ng/ml for 24 h. After the incubation, intracellular NO was measured by an OxiSelect Intacellular Nitric Oxide Assay Kit. BMDCs were stained with NO probe and the fluorescence was measured. The results shown are representative of two independent experiments. *P < 0.05; **P < 0.01; n.s., not significant, which is described in Materials and Methods.

Our previous study demonstrated that *A. faecalis*-derived LPS has low inflammatory activity (Shibata et al., 2018). DCs stimulated with *A. faecalis* LPS induced less IL-6 production than DCs stimulated with *E. coli* LPS (Shibata et al., 2018). NO production is known to be related to production of inflammatory cytokines, including IL-6, in DCs because these 2 processes are subject to similar transcriptional controls. Therefore, we examined effects of LPS on intracellular NO production in BMDCs. In the result, neither *A. faecalis* LPS nor *E. coli* LPS alone modulated the production of intracellular NO in BMDCs (Figure 2A). In addition, co-stimulation of BMDCs with both live *A. faecalis* and *A. faecalis* LPS did not influence the NO levels in these cells (Figure 2A), indicating that the LPS of *A. faecalis* does not upregulate intracellular NO production. In contrast, co-stimulation with live *A. faecalis* and *E. coli* LPS increased the production of intracellular NO in BMDCs (Figure 2A). We also examined the possibility that *A. faecalis* LPS could prevent live *E. coli* from inducing NO production. As a result, *A. faecalis* LPS did not affect live *E. coli*-induced NO production (Supplementary Figure 4). Additionally, we examined NO production in BMDCs stimulated with *A. faecalis* LPS or *E. coli* LPS in the presence of IFN-γ. Although both *A. faecalis* LPS and *E. coli* LPS induced intracellular NO production in a dose dependent manner, *A. faecalis* LPS was less active than *E. coli* LPS (Figure 2B). Collectively, these data show that LPS alone is not sufficient for the induction of intracellular NO production, however, LPS is one of the critical factors affecting the levels of intracellular NO production in BMDCs when considering the difference between *E. coli* and *A. faecalis*.

### Modulation of Energy Metabolism in DCs by *A. faecalis*

**FIGURE 3:**
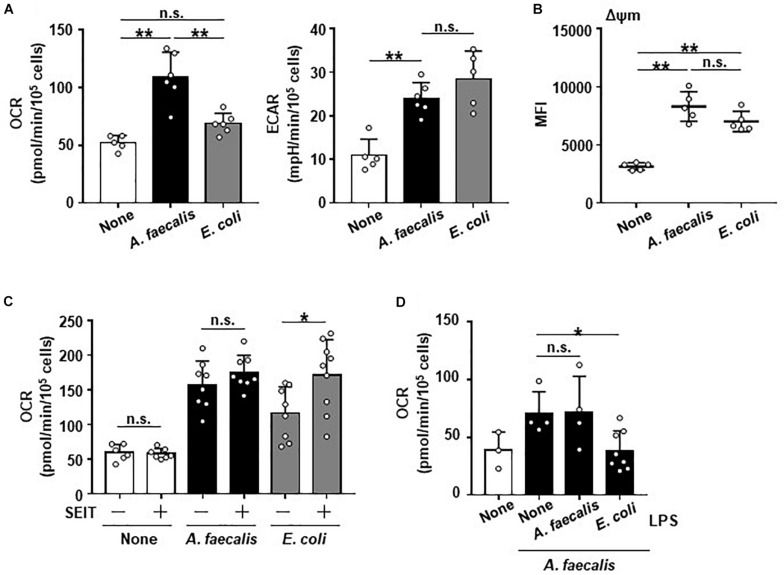
Unique alteration of the energy metabolism of DCs induced by *A. faecalis.* BMDCs were co-cultured without (None) or with live *A. faecalis* and *E. coli* at 10 MOI for 24 h. **(A)** After the co-culture, the basal oxygen consumption rate (OCR) and extracellular acidification rate (ECAR) were measured by using a flux analyzer. **(B)** After the co-culture, BMDCs were collected and stained with tetramethylrhodamine methyl ester and analyzed by flow cytometry to measure mitochondrial membrane potential (Δψm). **(C)** BMDCs were co-cultured without (None) or with live *A. faecalis* or *E. coli* at 10 MOI for 24 h in the absence or presence of the iNOS inhibitor SEIT and then OCR was measured by flux analyzer. **(D)** BMDCs were co-cultured without (None) or with live *A. faecalis* at 10 MOI for 24 h in the absence (None) or presence of *A. faecalis* LPS or *E. coli* LPS at 0.1 μg/ml and OCR was measured by flux analyzer. The results shown in **(A,C,D)** are combined from two independent experiment. The result shown in **(B)** is representative of two independent experiments. *P < 0.05; **P < 0.01; n.s., not significant, which is described in Materials and Methods.

It has been known that metabolic programming is associated with altering the activation and function of immune cells and that NO is involved in the regulation of energy metabolism and mitochondrial function (Erusalimsky and Moncada, 2007). Therefore, we examined the energy metabolic flux due to glycolysis and mitochondrial respiration in BMDCs after they were co-cultured with *A. faecalis* or *E. coli*. BMDCs carrying *E. coli* showed increased ECAR compared with non-stimulated DCs, but OCR was unchanged (Figure 3A), indicating that BMDCs carrying *E. coli* demonstrated Warburg metabolism characterized by shift to the glycolytic pathway (Pearce and Pearce, 2013). In contrast, although BMDCs carrying *A. faecalis*—like those with *E. coli*—had increased ECAR, OCR was higher in BMDCs carrying *A. faecalis* than in either non-stimulated BMDCs or BMDCs carrying *E. coli* (Figure 3A). These findings indicate the energy metabolism of BMDCs, especially mitochondrial respiration, differed depending on whether they took up *A. faecalis* compared with *E. coli*. Consistent with this result, mitochondrial membrane potentials were greater in BMDCs carrying *A. faecalis* than in non-stimulated BMDCs (Figure 3B). Unexpectedly, mitochondrial membrane potentials were similar between BMDCs carrying *E. coli* and those with *A. faecalis* (Figure 3B), suggesting that despite increases in mitochondrial membrane potentials, oxygen utilization was inhibited in the mitochondria of BMDCs carrying *E. coli*.

We then used the iNOS inhibitor SEIT to ask whether intracellular NO production affects energy metabolism of BMDCs. Treatment with SEIT decreased intracellular NO production in BMDCs carrying *E. coli* (Figure 1C). Treatment with SEIT did not affect OCR in non-stimulated BMDCs or BMDCs carrying *A. faecalis* and ECAR (Figure 3C and Supplementary Figure 5A). In contrast, exposure to SEIT increased OCR in BMDCs carrying *E. coli* (Figure 3C), suggesting that intracellular NO inhibited increase of OCR induced by stimulation with *E. coli* through competition with oxygen in mitochondrial respiration. When we examined the effects of LPS on mitochondrial respiration in BMDCs, we found that, consistent with effects on NO production (Figure 2), co-stimulation of BMDCs with both live *A. faecalis* and *E. coli* LPS decreased OCR compared with that in BMDCs carrying *A. faecalis* but unexposed to LPS (Figure 3D). In contrast, the OCR of BMDCs co-stimulated with live *A. faecalis* and *A. faecalis* LPS was increased and similar to that of BMDCs carrying *A. faecalis* but not treated with *E. coli* LPS (Figure 3D). Co-stimulation with live *A. faecalis* and *E. coli* LPS did not affect ECAR (Supplementary Figure 5B). Collectively, our results suggest that—due to the organism’s low LPS activity—DCs carrying *A. faecalis* maintain increased levels of mitochondrial respiration but do not produce NO.

### Less Induction of Apoptosis in DCs by *A. faecalis*

**FIGURE 4:**
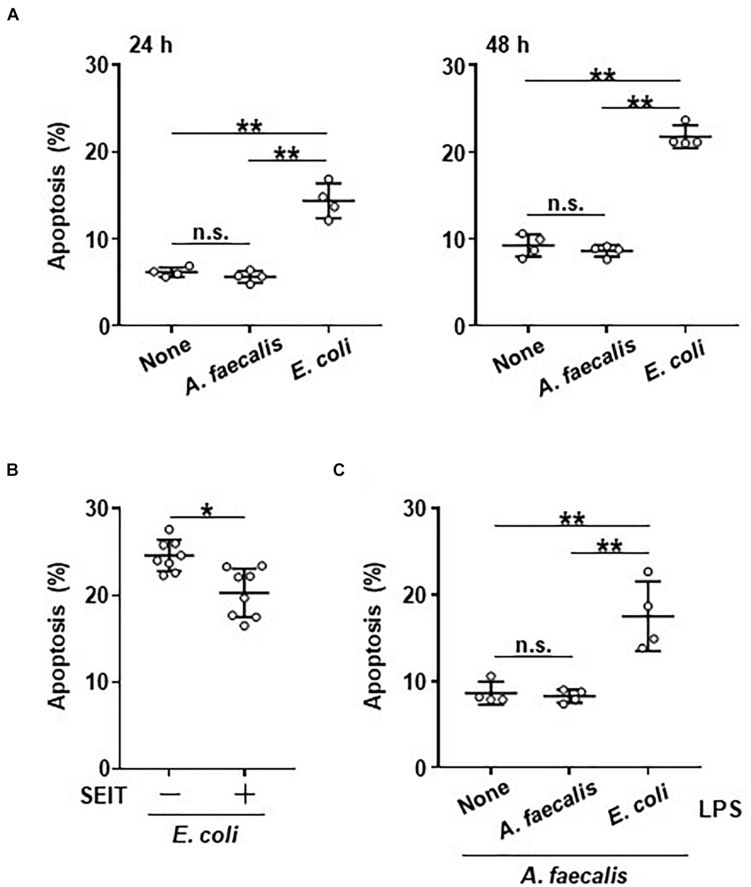
Little induction of apoptotic DCs by *A. faecalis.* **(A)** BMDCs were co-cultured without (None) or with live *A. faecalis* or *E. coli* at 10 MOI for 24 or 48 h, after which Annexin V^+^, 7AAD^–^ apoptotic cells were detected by FACS. **(B)** BMDCs were co-cultured with live *E. coli* at 10 MOI for 48 h in the absence or presence of iNOS inhibitor SEIT and then apoptotic cells were detected by FACS. **(C)** BMDCs were co-cultured with live *A. faecalis* at 10 MOI for 48 h in the absence (None) or presence of *A. faecalis* LPS or *E. coli* LPS at 0.1 μg/ml and then apoptotic cells were detected by FACS. The results shown in **(A,C)** are representative of two independent experiments. The result shown in **(B)** is combined from two independent experiments. *P < 0.05; **P < 0.01; n.s., not significant, which is described in Materials and Methods.

Mitochondrial activity is closely related to both cell survival and apoptosis (Green and Reed, 1998). Therefore, we examined the effects of bacterial uptake on apoptosis of BMDCs. The rate of apoptotic cells was similar between BMDCs carrying *A. faecalis* and non-stimulated BMDCs (Figure 4A). In contrast, the rate of apoptotic cells was higher in BMDCs carrying *E. coli* than in both non-stimulated BMDCs and BMDCs carrying *A. faecalis* (Figure 4A); this pattern suggests that DCs carrying *A. faecalis* can survive longer than DCs carrying *E. coli*. Next, we examined whether intracellular NO production affected apoptosis of BMDCs. Treatment with SEIT partially reduced the rate of apoptotic cells in BMDCs carrying *E. coli* (Figure 4B), indicating that intracellular NO production contributed to the induction of apoptosis in these cells but NO production does not seem to equate with apoptosis. Furthermore, the proportions of apoptotic cells were similar between *A. faecalis*-carrying BMDCs regardless of their exposure (or not) to *A. faecalis* LPS (Figure 4C). In contrast, co-stimulation with live *A. faecalis* and *E. coli* LPS increased BMDC apoptosis (Figure 4C). Collectively, these results suggest that *E. coli* induced the apoptosis of DCs through its high activity of LPS, whereas the low activity of *A. faecalis* LPS induced lower levels of apoptosis with little NO production.

## Discussion

In this study, we showed an intracellular symbiotic system involving lymphoid tissue–resident *Alcaligenes* that involves a unique energy metabolic shift in DCs. Unlike *E. coli*, *A. faecalis* did not induce intracellular NO production, thus enabling this organism to survive and persist in DCs as shown in our previous report (Fung et al., 2016). Consequently, LRCs including *A. faecalis* are thought to be able to coexist inside intestinal lymphoid tissue including PPs because these bacteria fail to stimulate the production of NO, which might otherwise lead to their elimination. Furthermore, DCs carrying *A. faecalis* had a low rate of apoptosis, which was associated with a unique energy metabolic shift that increased mitochondrial respiration. Considering that the presence of DCs is essential for the symbiosis of LRCs including *A. faecalis* in PPs (Fung et al., 2016; Shibata et al., 2018), the long-term survival of LRC-carrying DCs in the absence of apoptosis also contributes to the survival of these LRCs, including *A. faecalis*, in PPs. Therefore, LRCs including *A. faecalis* likely survive or persist in intestinal lymphoid tissues including PPs due to their ability to control NO production, energy metabolism, and apoptosis.

The low activity of *A. faecalis* LPS appears to associate with little induction of intracellular NO production in DCs carrying *A. faecalis*. NO is generated from L-arginine through the action of NOS enzymes. Whereas the constitutive forms of NOS, nNOS and eNOS, are present in neurons and endothelial cells, respectively, the inducible form, iNOS, is expressed in various cells including DCs and macrophages (Förstermann and Sessa, 2012). In macrophages, induction of iNOS requires co-stimulation by LPS and cytokines, such as IFN-γ. Specifically, LPS and IFN-γ activate the transcription factors nuclear factor κB and signal transducer and activator of transcription 1α to promote the expression of iNOS (Kleinert et al., 2004; Bogdan, 2015). Therefore, because the stimulation of iNOS expression requires LPS, DCs carrying *A. faecalis* did not produce intracellular NO due the lack of weakness of LPS activity. However, *A. faecalis*-harboring DCs retain the ability to produce cytokines, including IFN-γ, in light of the fact that co-stimulation with live *A. faecalis* and *E. coli* LPS led to NO production. In this regard, stimulation with DNA and RNA leads to IFN-γ production through TLR9 and TLR7 signaling pathways (Kawasaki and Kawai, 2014). Collectively, it is considered that DCs recognize non-commensal bacteria including *E. coli* because of their highly active LPS and other cellular components, such as DNA or RNA; in response to this recognition, DCs produce NO to eliminate these organisms. In contrast, LRCs including *A. faecalis* escape such elimination because of the low activity of their LPS.

In addition to the low activity of LPS in the induction of iNOS expression, *A. faecalis* seems to possess NO degrading activity. Indeed, *A. faecalis* weakly induced iNOS expression, but no elevation of intracellular NO was detected in BMDCs carrying *A. faecalis*. Genomic analysis has indicated that *A. faecalis* possesses *NorB* gene encoding NO reductase to reduce NO (Braker and Tiedje, 2003; Obata et al., 2010). Thus, it is considered that NO was not elevated in DCs carrying *A. faecalis* due to the low activity of LPS in the induction of iNOS expression together with a potential ability to degrade NO. In spite of the NO degrading activity of *A. faecalis*, *E. coli* LPS induced NO production in the presence of *A. faecalis*. Simple explanation is that the amount of NO induced by *E. coli* LPS plus *A. faecalis* exceeded the capacity of *A. faecalis* to degrade it. The second possibility is the influence of environmental changes induced by inflammation. For example, it was reported that the activity of NO reductase is influenced by pH (Iwamoto et al., 2001). Since inflammatory activation of DCs is associated with the reduction of pH caused by lactic acidosis (Erra Díaz et al., 2018), *E. coli* LPS-mediated inflammatory environment might decrease the NO degrading activity of *A. faecalis*. Additionally, low pH has been shown to induce the expression of iNOS through NF-κB activation (Erra Díaz et al., 2018).

NO competes with oxygen to inhibit the mitochondrial enzyme cytochrome oxidase (complex IV) (Brown and Cooper, 1994). Furthermore, inhibition of complex IV by continuous exposure to NO induces the development of oxidative stress with the subsequent inhibition of other mitochondrial enzymes (Clementi et al., 1998; Beltrán et al., 2002). Various stimuli including oxidants can trigger mitochondria to release caspase-activating proteins, such as cytochrome C and apoptosis-inducing factor (AIF), which in turn activate caspases including caspase 9 and result in apoptosis (Jiang and Wang, 2004; Wang and Youle, 2009). Thus, NO inhibits mitochondrial respiration and subsequently induces apoptosis. Therefore, in the current study, DCs carrying *A. faecalis* showed lower rates of apoptotic cells and increased levels of OCR, compared with DCs carrying *E. coli*. The results of the experiments using an iNOS inhibitor and LPS supported these findings. When considering the link between apoptosis and NO production, unlike stimulation with LPS alone, co-stimulation with IFN-γ and LPS has been known to induce NO-mediated apoptosis in DCs, especially iNOS-positive one (Lu et al., 1996) and the mitochondrial cytochrome C release is recognized as an underlying mechanism (Ranjan et al., 2004). However, treatment with an iNOS inhibitor led to equivalent OCRs between DCs regardless of the bacteria that the cells carried, whereas this treatment only partially inhibited apoptosis of DCs carrying *E. coli*. In this regard, diverse stimuli induce apoptosis through both the intrinsic pathway (also called the mitochondrial pathway) and the extrinsic pathway (Elmore, 2007). For example, tumor necrosis factor α, a pro-inflammatory cytokine, leads to apoptosis through activation of caspase 8 via the tumor necrosis factor receptor (Elmore, 2007). Some bacteria, such as *Streptococcus pneumoniae*, can induce apoptosis in DCs through the production of bacterial toxins, including pneumolysin; this effect is thought to facilitate bacterial invasion by impairing the antibacterial immune responses (Colino and Snapper, 2003). Indeed, *A. faecalis* shows low inflammatory properties, including less production of TNF-α from BMDCs in comparison with *E. coli* (Fung et al., 2016; Shibata et al., 2018). Therefore, perhaps *E. coli* induces apoptosis in DCs through both NO-dependent and -independent mechanisms. Although further experiments are necessary to fully understand the underlying mechanisms including the role of LPS in the induction of NO and apoptosis, in any case, we consider that LRCs including *A. faecalis* either actively suppress or at least do not stimulate apoptotic pathways, so that the survival of DCs carrying LRCs is prolonged, perhaps thus contributing to the establishment of a symbiotic relationship between LRCs and DCs.

One of the most interesting findings in this study is that DCs carrying *A. faecalis* showed increased mitochondrial respiration as well as mitochondrial membrane potentials. In general, activated DCs that are stimulated with the TLR4 ligand LPS show increased glycolysis and decreased mitochondrial respiration, which are related to the accumulation of citrate and succinate and lead to the generation of NO and IL-1β (Mills and O’Neill, 2016; Escoll and Buchrieser, 2018). Therefore, it has been thought that a metabolic shift to glycolysis was necessary for cytokine production and protection against bacteria in DCs. In contrast, the TLR2 ligand P3C induces increased both glycolysis and mitochondrial respiration in monocytes, thus activating host defense mechanisms, including cytokine production and phagocytosis (Lachmandas et al., 2016). Therefore, the metabolic responses of immune cells to bacterial components vary by cell type and bacterial species. In this context, it is very interesting to see that DCs carrying live *A. faecalis* exhibited a different metabolic shift than those stimulated by *E. coli* cells or by LPS only. Moreover, given that DCs play important roles in the maintenance of immunologic homeostasis, including protective immune responses to pathogens and tolerance to food antigens and commensal bacteria in the intestine (Kayama and Takeda, 2012; Bekiaris et al., 2014), this unique metabolic shift in DCs may contribute to the creation of a specialized environment in the intestine. The mechanism by which *A. faecalis* increases mitochondrial respiration and the immunologic function of this unique metabolic shift remain unclear, and further investigation is needed to fully understand the immunologic significance of LRC-induced metabolic changes in intestinal lymphoid tissue.

## Conclusion

In conclusion, LRCs including *A. faecalis* establish a symbiotic relationship with DCs in intestinal lymphoid tissue because these organisms elicit negligible amounts of NO due to the low activity of LPS as one of the underlying mechanisms. Furthermore, this symbiosis induces unique metabolic changes that are associated with cell physiology, such as apoptosis, in DCs.

## Data Availability Statement

The raw data supporting the conclusions of this article will be made available by the authors, without undue reservation.

## Ethics Statement

The animal study was reviewed and approved by the Animal Care and Use Committee of the National Institutes of Biomedical Innovation, Health, and Nutrition (approval no. DS27-48R10).

## Author Contributions

KH designed the study, performed experiments, analyzed the data, and wrote the manuscript. NS, AS, TU, YT, and TNi performed the experiments. TNa, HT, KF, and HK provided helpful discussion. JK designed the study and wrote the manuscript. All authors approved the final version of the manuscript.

## Conflict of Interest

The authors declare that the research was conducted in the absence of any commercial or financial relationships that could be construed as a potential conflict of interest.

## Supplementary Material

Supplementary Figure 1 Similar changes in markers for phagocytosis and activation of BMDCs induced by *A. faecalis* or *E. coli.* BMDCs were co-cultured without (None) or with live *A. faecalis* or *E. coli* at 10 MOI for 24 h. **(A)** *Rab7* and *Lamp-1* gene expression were measured by RT-qPCR. **(B)** MHCII expression was measured by FACS. The results shown are representative of two independent experiments.

Supplementary Figure 2 FACS histogram of iNOS expression. As shown in Figure 1B, BMDCs were co-cultured without (None) or with live *A. faecalis* or *E. coli* at 10 MOI for 24 h and then iNOS expression was measured by intracellular FACS. The representative histograms are shown.

Supplementary Figure 3 Degrading of NO by *A. faecalis. A. faecalis* (1 × 10^8^, 3 × 10^8^, 1 × 10^9^ CFU/well) was incubated with 100 μM NOR3, a NO inducing reagent, at 37°C for 1 h. After the incubation, the concentration of NO was measured by Griess reagent kit. The results shown are representative of two independent experiments.

Supplementary Figure 4 BMDCs were co-cultured without (None) or with live *E. coli* at 10 MOI in the absence (None) or presence of *A. faecalis* LPS at 0.1 μg/ml for 24 h, after which intracellular NO was measured by an OxiSelect Intracellular Nitric Oxide Assay Kit. BMDCs were stained with NO probe and the fluorescence was measured. The results shown are representative of two independent experiments.

Supplementary Figure 5 Little influence of treatment of SEIT or LPS on ECAR of BMDCs carrying *A. faecalis* or *E. coli.* **(A)** BMDCs were co-cultured without (None) or with live *A. faecalis* or *E. coli* at 10 MOI for 24 h in the absence or presence of the iNOS inhibitor SEIT and then extracellular acidification rate (ECAR) was measured by flux analyzer. **(B)** BMDCs were co-cultured without (None) or with live *A. faecalis* at 10 MOI for 24 h in the absence (None) or presence of *A. faecalis* LPS or *E. coli* LPS at 0.1 μg/ml and ECAR was measured by flux analyzer. The results shown are combined from two independent experiments.
