# Supplementary material for: Lymphoid Tissue–Resident Alcaligenes Establish an Intracellular Symbiotic Environment by Creating a Unique Energy Shift in Dendritic Cells
Source: Front Microbiol. 2020 Sep 24;11:561005. doi: 10.3389/fmicb.2020.561005 (PMC7545135; doi:10.3389/fmicb.2020.561005)
Supplement: Supplementary file 1 [file Data_Sheet_1.PDF]

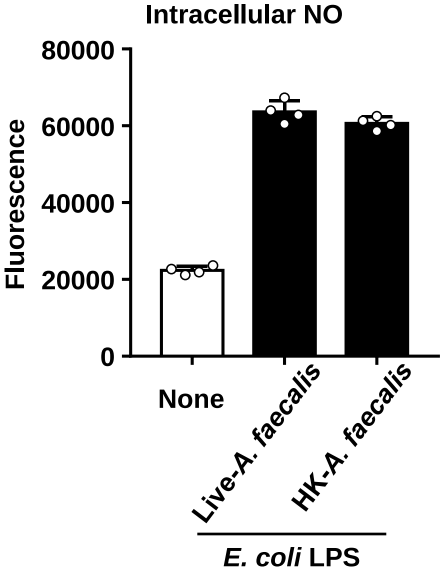

Appendix data 1

BMDCs were co-cultured without (None) or with live *A. faecalis* or heat-killed *A. faecalis* (HK- *A. faecalis*) at 10 MOI in the presence of *E. coli* LPS at 0.1 µg/ml for 24 h. After the co-culture, intracellular NO was measured by using an OxiSelect Intacellular Nitric Oxide Assay Kit (Cell Biolabs). BMDCs were stained with NO probe and the fluorescence was measured.

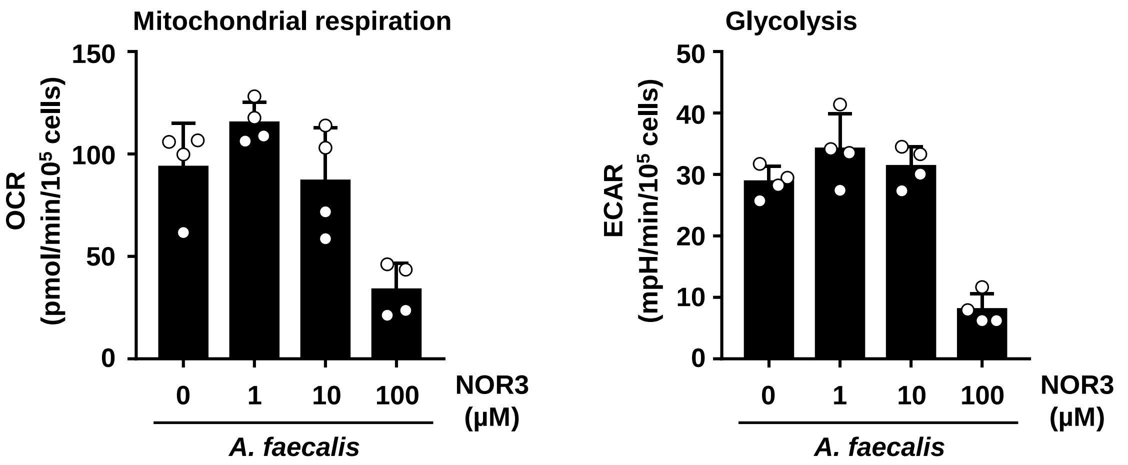

Appendix data 2

BMDCs were co-cultured with live *A. faecalis* at 10 MOI in the absence or presence of NOR3, a NO inducing reagent, at 1-100 µM for 24 h. After the co-culture, the basal oxygen consumption rate (OCR) and extracellular acidification rate (ECAR) were measured by using a flux analyzer.

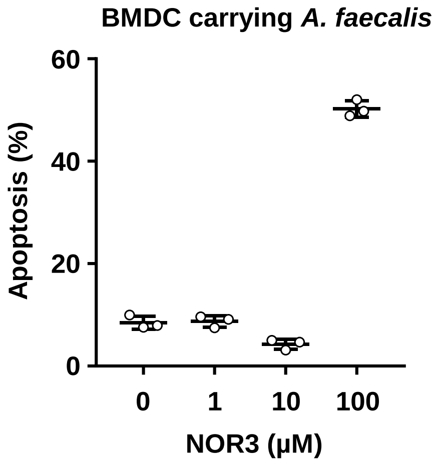

Appendix data 3

BMDCs were co-cultured with live *A. faecalis* at 10 MOI in the absence or presence of NOR3, a NO inducing reagent, at 1-100 μM for 48 h, after which Annexin V^+, $7AAD^-$ apoptotic cells were detected by FACS.
