# Supplementary figures and images for: Lymphoid Tissue–Resident Alcaligenes Establish an Intracellular Symbiotic Environment by Creating a Unique Energy Shift in Dendritic Cells
Source: Front Microbiol. 2020 Sep 24;11:561005. doi: 10.3389/fmicb.2020.561005 (PMC7545135; doi:10.3389/fmicb.2020.561005)

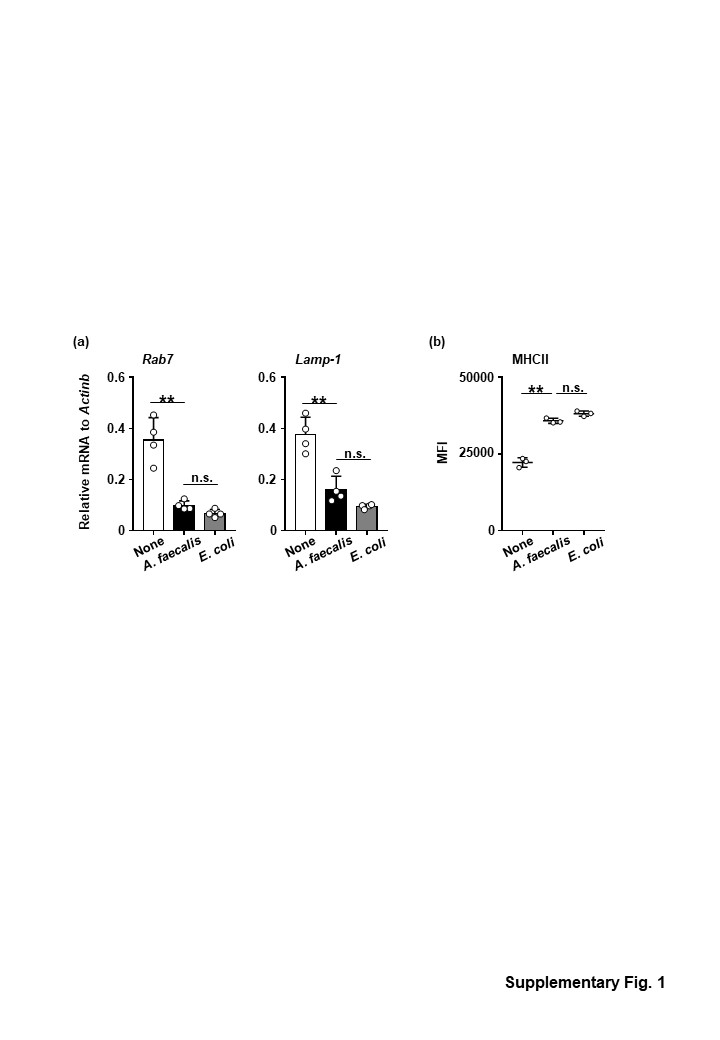

Supplement: Supplementary Figure 1 — Similar changes in markers for phagocytosis and activation of BMDCs induced by A. faecalis or E. coli. BMDCs were co-cultured without (None) or with live A. faecalis or E. coli at 10 MOI for 24 h. (A) Rab7 and Lamp-1 gene expression were measured by RT-qPCR. (B) MHCII expression was measured by FACS. The results shown are representative of two independent experiments. [file Data_Sheet_2.zip › Supplementary Figures/SupplementaryFig1.JPG]

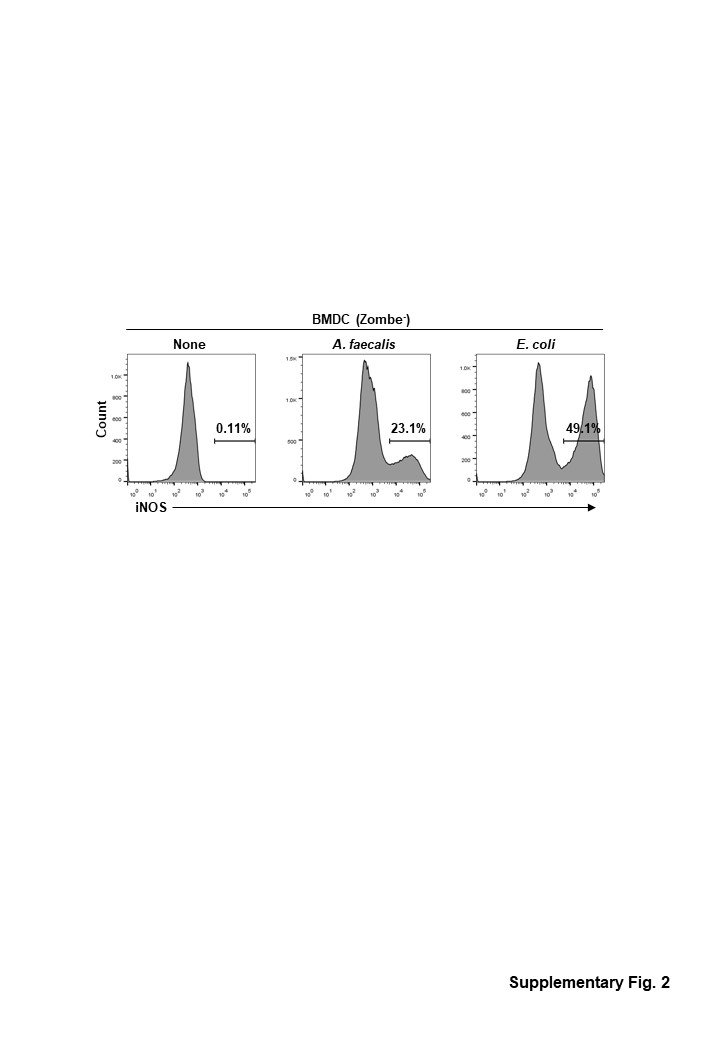

Supplement: Supplementary Figure 1 — Similar changes in markers for phagocytosis and activation of BMDCs induced by A. faecalis or E. coli. BMDCs were co-cultured without (None) or with live A. faecalis or E. coli at 10 MOI for 24 h. (A) Rab7 and Lamp-1 gene expression were measured by RT-qPCR. (B) MHCII expression was measured by FACS. The results shown are representative of two independent experiments. [file Data_Sheet_2.zip › Supplementary Figures/SupplementaryFig2.JPG]

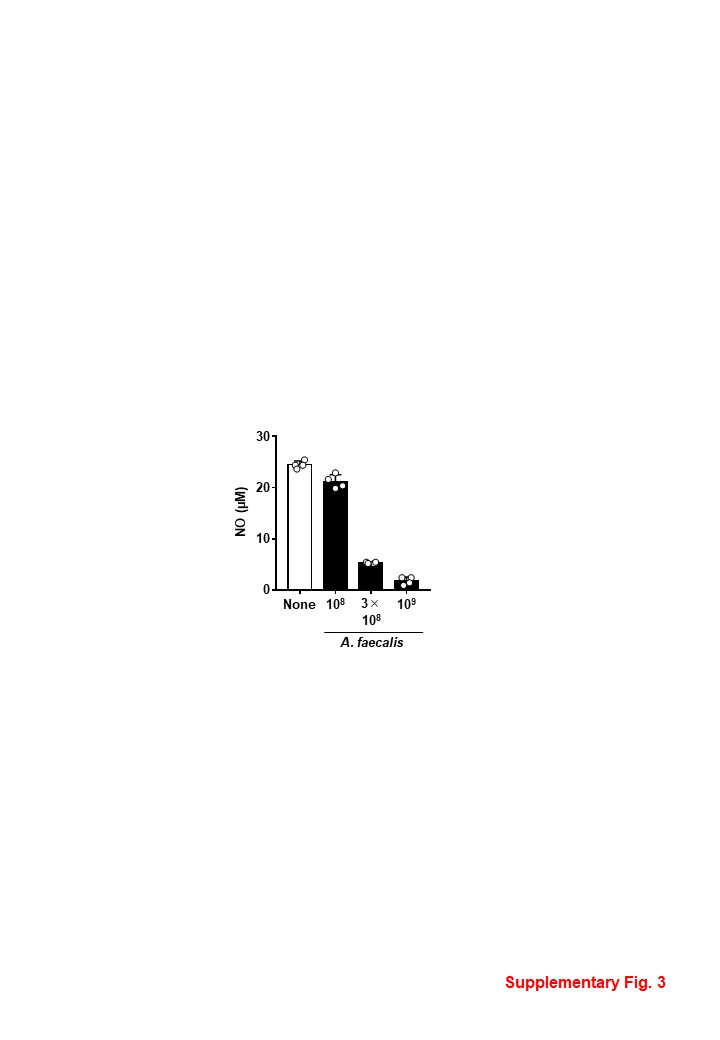

Supplement: Supplementary Figure 1 — Similar changes in markers for phagocytosis and activation of BMDCs induced by A. faecalis or E. coli. BMDCs were co-cultured without (None) or with live A. faecalis or E. coli at 10 MOI for 24 h. (A) Rab7 and Lamp-1 gene expression were measured by RT-qPCR. (B) MHCII expression was measured by FACS. The results shown are representative of two independent experiments. [file Data_Sheet_2.zip › Supplementary Figures/SupplementaryFig3.JPG]

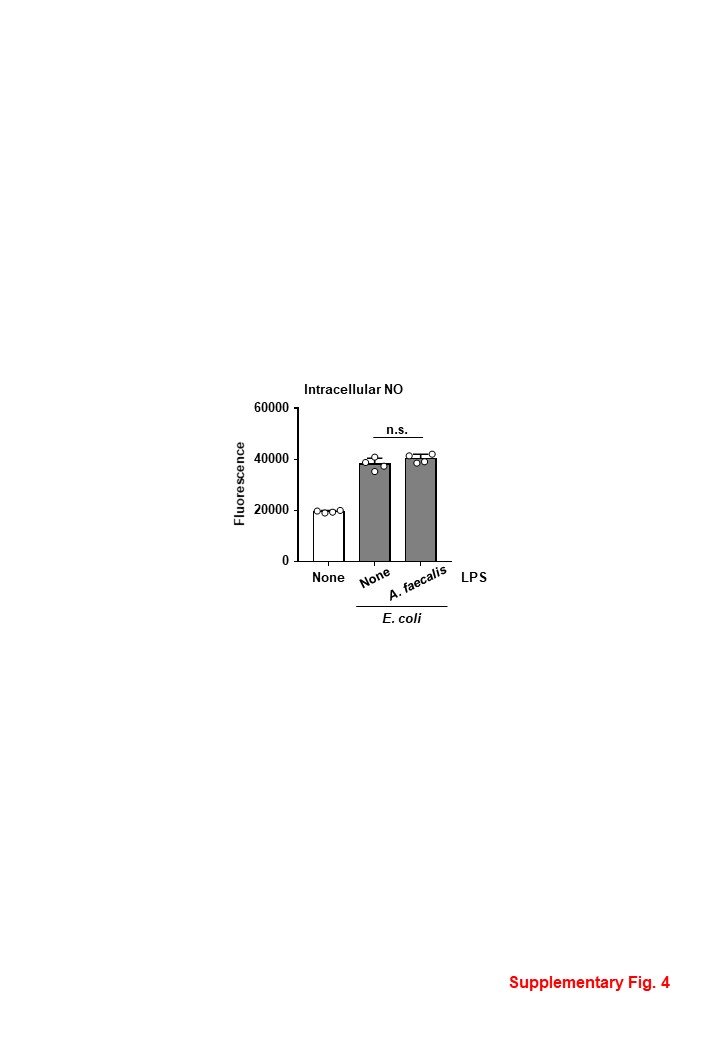

Supplement: Supplementary Figure 1 — Similar changes in markers for phagocytosis and activation of BMDCs induced by A. faecalis or E. coli. BMDCs were co-cultured without (None) or with live A. faecalis or E. coli at 10 MOI for 24 h. (A) Rab7 and Lamp-1 gene expression were measured by RT-qPCR. (B) MHCII expression was measured by FACS. The results shown are representative of two independent experiments. [file Data_Sheet_2.zip › Supplementary Figures/SupplementaryFig4.JPG]

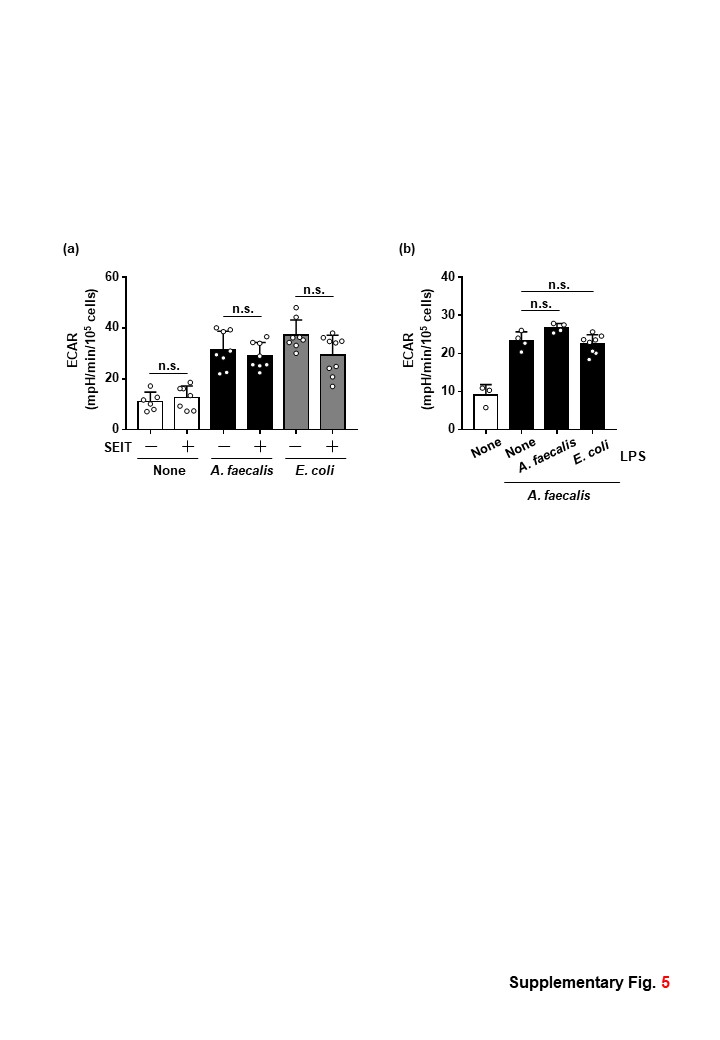

Supplement: Supplementary Figure 1 — Similar changes in markers for phagocytosis and activation of BMDCs induced by A. faecalis or E. coli. BMDCs were co-cultured without (None) or with live A. faecalis or E. coli at 10 MOI for 24 h. (A) Rab7 and Lamp-1 gene expression were measured by RT-qPCR. (B) MHCII expression was measured by FACS. The results shown are representative of two independent experiments. [file Data_Sheet_2.zip › Supplementary Figures/SupplementaryFig5.JPG]
